# Cumulative subgroup analysis to reduce waste in clinical research for individualised medicine

**DOI:** 10.1186/s12916-016-0744-x

**Published:** 2016-12-15

**Authors:** Fujian Song, Max O. Bachmann

**Affiliations:** Norwich Medical School, Faculty of Medicine and Health Science, University of East Anglia, Research Park, Norwich, Norfolk NR4 7TJ UK

**Keywords:** Subgroup analysis, Individual patient data, Cumulative meta-analysis, Randomised controlled trials, Individualised medicine

## Abstract

**Background:**

Although subgroup analyses in clinical trials may provide evidence for individualised medicine, their conduct and interpretation remain controversial.

**Methods:**

Subgroup effect can be defined as the difference in treatment effect across patient subgroups. Cumulative subgroup analysis refers to a series of repeated pooling of subgroup effects after adding data from each of related trials chronologically, to investigate the accumulating evidence for subgroup effects. We illustrated the clinical relevance of cumulative subgroup analysis in two case studies using data from published individual patient data (IPD) meta-analyses. Computer simulations were also conducted to examine the statistical properties of cumulative subgroup analysis.

**Results:**

In case study 1, an IPD meta-analysis of 10 randomised trials (RCTs) on beta blockers for heart failure reported significant interaction of treatment effects with baseline rhythm. Cumulative subgroup analysis could have detected the subgroup effect 15 years earlier, with five fewer trials and 71% less patients, than the IPD meta-analysis which first reported it. Case study 2 involved an IPD meta-analysis of 11 RCTs on treatments for pulmonary arterial hypertension that reported significant subgroup effect by aetiology. Cumulative subgroup analysis could have detected the subgroup effect 6 years earlier, with three fewer trials and 40% less patients than the IPD meta-analysis. Computer simulations have indicated that cumulative subgroup analysis increases the statistical power and is not associated with inflated false positives.

**Conclusions:**

To reduce waste of research data, subgroup analyses in clinical trials should be more widely conducted and adequately reported so that cumulative subgroup analyses could be timely performed to inform clinical practice and further research.

**Electronic supplementary material:**

The online version of this article (doi:10.1186/s12916-016-0744-x) contains supplementary material, which is available to authorized users.

## Background

Randomised controlled trials (RCTs) provide the most valid evidence on effects of treatments and healthcare interventions, and results of RCTs are usually reported as estimated average effects. However, average effects from RCTs may not be generalisable to individual patients in clinical practice, because of heterogeneity across individual patients in terms of demographic characteristics, genetic features, disease severity, co-morbidities and other factors [1, 2]. Subgroup analysis is often used in RCTs to investigate differences in treatment effect between patients with different characteristics [3]. A study found that subgroup analyses were reported in 44% of the 469 RCTs published in core clinical journals in 2007 [4].

There are well known limitations with subgroup analyses in RCTs, including possible false positive subgroup effects due to multiple testing, and false negative subgroup results due to inadequate statistical power [5]. It has been recommended that only a small number of pre-specified subgroup analyses in RCTs should be conducted using appropriate statistical methods [6, 7]. Methods have been recommended for assessing credibility of subgroup analyses in RCTs [8, 9]. However, the conduct, reporting and interpretation of subgroup analysis in RCTs are still controversial [10, 11].

Although meta-analyses of RCTs are generally accepted for estimating the overall treatment effects, subgroup analyses in meta-analysis are hampered because of inadequate data on subgroups in published trials [12]. Meta-analysis using individual patient data (IPD) has been increasingly used to conduct more statistically powerful subgroup analyses by pooling data from multiple trials [13]. A recent study found that significant subgroup effects were identified in 44 IPD meta-analyses and in only three aggregate data meta-analyses among 204 paired IPD meta-analyses and aggregate data meta-analyses [14].

Previous studies regarding subgroup analysis methodology have focused mainly on the results of individual trials, and research data may have been wasted for detecting clinically important differences in treatment effects between patient subgroups. The objective of the current study is to illustrate the usefulness and statistical properties of cumulative subgroup analysis by providing empirical evidence from case studies and computing simulations.

## Methods

Patient characteristics at baseline are relevant subgroup variables in the current study, although subgroup analyses may also be conducted by other types of variables. Subgroup effect in a trial is defined as the interaction of treatment with a subgroup variable, or the difference in treatment effect across subgroups [5]. In the current study, subgroup effect is measured by the ratio of relative treatment effects between subgroups (e.g. ratio of odds ratios, ratio of hazard ratios) for binary outcomes, and the difference in absolute mean differences or standardised mean differences between subgroups for continuous outcomes.

### Cumulative subgroup analysis

For estimating overall treatment effects, cumulative meta-analysis consists of a series of repeated meta-analyses after adding data from each new trial chronologically [15]. It can be used to reveal the contribution of individual trials to the overall estimate, and to identify the earliest time at which the pooled effect becomes statistically significant. We applied the method of cumulative meta-analysis to investigate changes in estimates of subgroup effects over time. Statistical methods for cumulative subgroup analysis are presented in Additional file 1.

### Case studies

We searched PubMed to identified recently published IPD meta-analyses with sufficient data to perform cumulative subgroup analysis according to year of publication (see Additional file 2 for search strategy). Two cases were presented in detail to illustrate the usefulness of cumulative subgroup analysis. The first case study used data from an IPD meta-analysis of beta blockers for heart failure [16], and the second case study used data from an IPD meta-analysis that compared treatment effects for connective tissue disease-associated pulmonary arterial hypertension (PAH) and for idiopathic PAH [17]. Data from the two case studies were used to conduct cumulative subgroup analyses. The relevant subgroup analyses in publications of clinical trials and aggregate data meta-analyses were also examined, and compared with the results of the IPD meta-analyses.

### Computer simulations

Computer simulations were conducted to assess true and false positive rates of conventional and cumulative subgroup analyses in trials using different input parameters in terms of the assumed subgroup effects and sample size (see Additional file 3 for simulation methods and input values). A series of sequentially ordered RCTs were simulated. For each of the simulated trials, the computer programme randomly generated individual patients according to distributions of assumed values of input parameters, and the simulated individual patients were randomly allocated to a control and a treatment arm. Simulated patients belong to different subgroups according to four independent characteristics (X_1_, X_2_, X_3_ and X_4_) at baseline, and subgroup analyses were conducted in each simulated trial by these baseline characteristics. It is assumed that only X_1_ is an effect modifier, and the other three factors (X_2_, X_3_, and X_4_) are not truly associated with the treatment effect. The treatment effect for patients with X_1_ (i.e. X_1_ = 1) is greater than patients without X_1_ (i.e. X_1_ = 0). The simulated subgroup effect is measured by ratio of odds ratios (ROR), which is assumed to be ROR less than 1.0 for patients with X_1_ = 1 versus X_1_ = 0, and ROR = 1.0 for the same comparisons of patients for X_2_, X_3_ and X_4_. For example, if ROR = 0.8 for patients with X_1_ and OR = 0.7 for patients without X_1_, the treatment effect for patients with X_1_ will be OR = 0.7 × 0.8 = 0.56. We used logistic regression model with an interaction term to conduct subgroup analyses in the simulated trials. In the simulation we calculated *P* values for subgroup effect each time a new trial was added to the analysis, and the observation of any statistically significant subgroup effect was not used to stop the cumulative subgroup analysis early before all relevant trials were included. For each scenario, 5000 replications were simulated to estimate the statistical power and the rate of type I error in subgroup analyses.

Data analyses and computer simulations were performed using Stata/IC 13 and R language [18]. Statistical significance was defined as two-sided *P* ≤ 0.05.

## Results

### Case study 1: beta blockers for heart failure

**Fig. 1:**
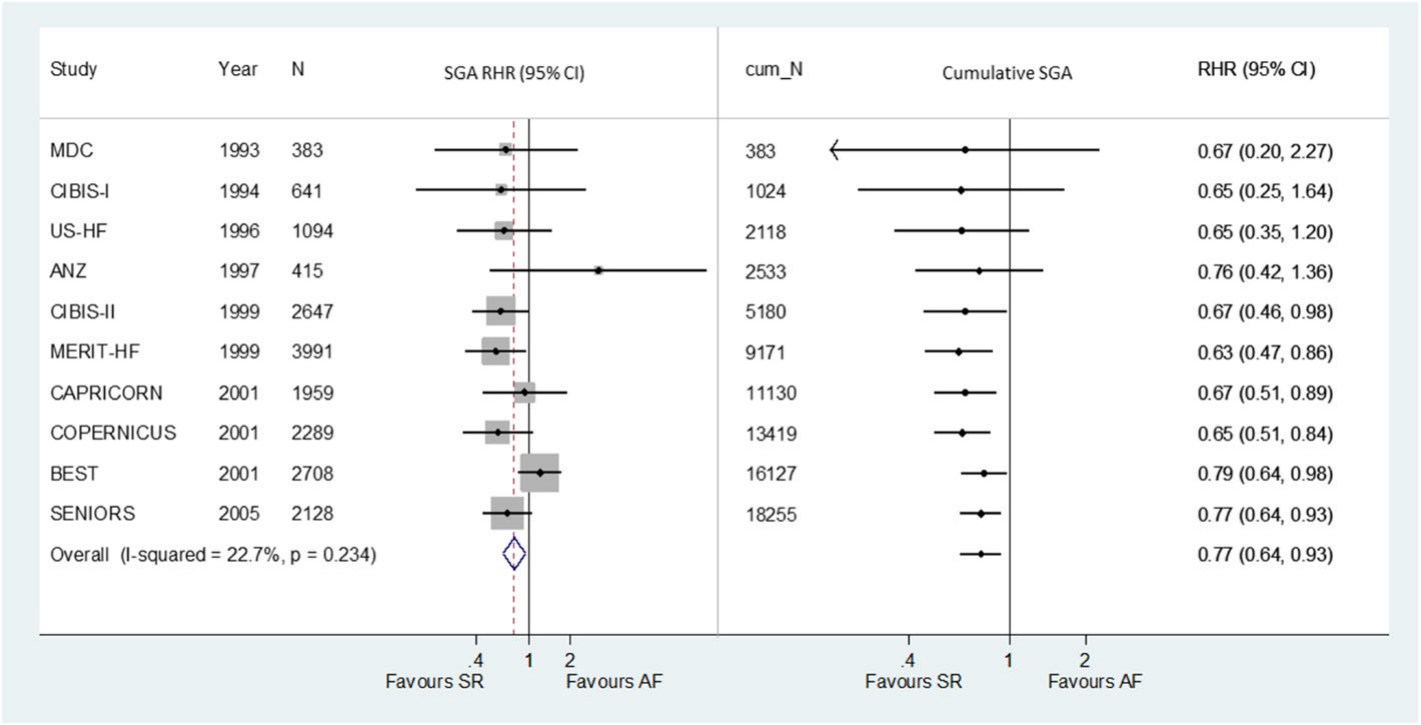
Beta blockers for heart failure: meta-analysis and cumulative subgroup analysis for differences in treatment effect between patients with sinus rhythm (SR) and those with atrial fibrillation (AF) at baseline

Published in 2014, an IPD meta-analysis of 10 RCTs found that the use of beta blockers reduced all-cause mortality for patients with heart failure and sinus rhythm, but not for those with heart failure plus atrial fibrillation at baseline [16]. The subgroup effect was statistically significant (*P* = 0.002 for interaction with baseline rhythm). Using reported hazard ratios for patients with sinus rhythm and those with atrial fibrillation, we estimated the ratio of hazard ratios (RHR) between the two subgroups in each of the 10 RCTs (Fig. 1). There was no statistically significant heterogeneity in RHRs across RCTs (I^2^ = 22.7%, *P* = 0.23), with a pooled RHR of 0.77 (95% CI, 0.64–0.93; *P* = 0.007). Results of cumulative subgroup analysis revealed that the subgroup effect reached statistical significance by 1999 (pooled RHR = 0.67; 95% CI, 0.46–0.98; *P* < 0.05), when five RCTs had been published with a total of 5180 patients. The overall subgroup effect had not been materially changed since 1999, with five more RCTs and 13,075 more patients (Fig. 1). That is, the significant subgroup effect could have been detected in cumulative subgroup analysis 15 years earlier and with 71.6% less patients than the IPD meta-analysis published in 2014 [16] which first reported it.

Subgroup analysis stratified by baseline rhythm was not reported in publications of the main results in the 10 RCTs [19–28]. After the publication of the main results, subgroup results by cardiac rhythm at baseline were presented in subsequent reports of the four trials [29–32]. The first was published in 2001 [32], and found statistically significant interaction between treatment and rhythm (*P* < 0.01) using data from the CIBIS-II trial [23]. Although there were numerous aggregate data meta-analyses on beta blockers for heart failure, we identified only one meta-analysis, published in 2013, that performed subgroup analysis by baseline rhythm [33], and which included published data from four trials [29–32].

### Case study 2: treatment for pulmonary arterial hypertension

**Fig. 2:**
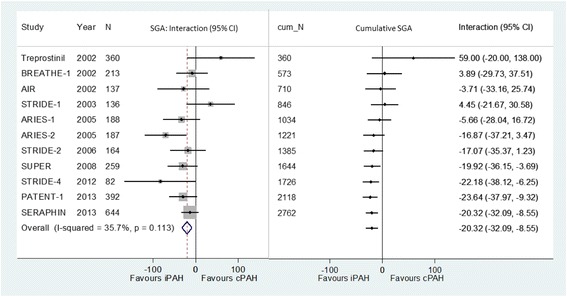
Meta-analysis and cumulative subgroup analysis of differences in treatment effect between idiopathic (iPAH) and connective tissue disease-associated pulmonary arterial hypertension (cPAH)

An IPD meta-analysis included 11 placebo-controlled RCTs that evaluated drugs for pulmonary arterial hypertension (PAH) [17]. For the improvement in 6-minute walk distance, the IPD meta-analysis found that treatment for connective tissue disease-associated PAH was less effective than for idiopathic PAH (Fig. 2). Using data from this IPD meta-analysis, cumulative subgroup analysis revealed that the interaction between treatment and diagnosis became statistically significant (*P* < 0.05) by 2008, when eight RCTs involving a total of 1644 patients had been published (Fig. 2). The significant subgroup effect was detectable 6 years earlier, and with 1118 less patients, than the IPD meta-analysis which first reported it.

Although subgroup analysis by aetiology was mentioned in most of the included trials, no significant subgroup effect by diagnosis was reported in original publications of the included RCTs [34–43]. For example, data from the ARIES-2 trial showed a statistically significant subgroup effect (Fig. 2), which was not reported in the original publication of the trial [40]. Subgroup analysis by aetiology was not conducted in any of aggregate data meta-analyses of trials that recruited patients with both connective tissue disease-associated PAH and idiopathic PAH [44–47].

### Results of computer simulations

Table 1 shows rates of true and false positive subgroup effects (*P* ≤ 0.05) observed in simulated trials under various scenarios. As expected, the true positive rates (statistical power) are associated with the subgroup effect (ROR), sample size, event rate in the control arm, and the proportion of patients in the X_1_ subgroup. The false positive rates (type I error) for each of the three variables (X_2_, X_3_ and X_4_) are about 5%, corresponding to the definition of statistical significance used (*P* ≤ 0.05). However, the false positive rate will be inflated (to about 14.3%) if it is calculated by identifying at least one false positive subgroup effect among all subgroup analyses by X_2_, X_3_ and X_4_.

**Table 1:** True and false positive rates of significant subgroup effects in simulated trials under different scenarios

Variables	Input values	True positive	False positive			
		X_1_	X_2_	X_3_	X_4_	Any of X_2_, X_3_, X_4_
Ratio of odds ratios (ROR)	0.90	6.2%	4.9%	5.0%	5.0%	14.2%
	0.80	10.3%	5.0%	5.1%	4.9%	14.2%
	0.70	17.9%	4.8%	5.0%	5.0%	14.1%
No. of patients per arm	200	7.5%	4.9%	4.9%	5.1%	14.1%
	400	10.1%	5.0%	5.0%	4.8%	14.2%
	600	12.6%	5.1%	5.0%	4.8%	14.2%
Event rate in the control arm	0.20	8.7%	4.9%	5.1%	5.0%	14.3%
	0.30	10.2%	5.2%	5.1%	5.1%	14.6%
	0.40	11.3%	5.1%	4.9%	5.1%	14.4%
Proportion of patients belonging to a subgroup	20%	8.0%	4.6%	5.0%	4.9%	14.2%
	30%	9.3%	5.1%	4.9%	4.9%	14.4%
	40%	10.0%	5.3%	4.9%	5.1%	14.5%

Except for varying input values shown in the table, the following basic input parameters were used for other variables: No. of trials = 10; No. of patients per arm = 400; OR = 0.7; event rate in the control arm = 0.3; heterogeneity variance = 0.01; ROR = 0.8; proportion of patients with X_1_ = 0.4. Positive rates were calculated based on 5000 simulations for each scenario (with a given set of input parameters)

Results of the cumulative subgroup analyses that used data from simulated trials are shown in Table 2. Given the assumed subgroup effect and sample size, the true positive rate was only about 10% with a single trial, 15% with two trials, and increased to about 80% with 18 trials (Table 2). For individual variables that are not associated with the treatment effect, the false positive rates in cumulative subgroup analyses were about 5%, corresponding to the defined statistical significance at *P* ≤ 0.05 (Table 2).

**Table 2:** True and false positive rates in cumulative subgroup analysis using data from simulated trials

No. of trials sequentially included	No. of total patients involved	True positive	False positive		
		X1	X2	X3	X4
1	800	9.8%	4.8%	5.4%	5.2%
2	1600	14.8%	4.7%	5.3%	4.5%
3	2400	19.7%	5.1%	5.5%	5.0%
4	3200	25.7%	5.1%	5.5%	5.3%
5	4000	30.7%	4.9%	5.4%	4.6%
6	4800	36.4%	5.0%	5.3%	4.9%
7	5600	41.3%	5.4%	5.5%	4.6%
8	6400	46.0%	5.5%	5.2%	4.7%
9	7200	50.7%	5.5%	5.4%	4.5%
10	8000	54.4%	5.2%	5.2%	4.9%
11	8800	58.3%	5.5%	5.3%	4.9%
12	9600	61.6%	5.2%	4.9%	4.5%
13	10,400	65.2%	5.2%	5.3%	4.7%
14	11,200	68.9%	5.5%	5.3%	4.9%
15	12,000	71.9%	5.3%	5.5%	4.9%
16	12,800	74.4%	5.5%	5.4%	4.6%
17	13,600	77.1%	5.7%	5.5%	4.5%
18	14,400	79.6%	5.7%	5.1%	4.8%
19	15,200	82.0%	5.9%	4.7%	4.7%
20	16,000	83.8%	5.7%	4.6%	4.7%

Except for varying input values shown in the table, the following basic input parameters were used for other variables: No. of patients per arm = 400; OR = 0.7; event rate in the control arm = 0.3; heterogeneity variance = 0.01; ROR = 0.8; proportion of patients with X_1_, X_2_, X_3_ and X_4_ = 0.4. Positive rates were based on 5000 simulations

## Discussion

Although subgroup analyses in randomised trials have well-known limitations, such as inadequate statistical power and inflated false positive rate, identification of subgroup effects is important for clinical practice and further research. The results of the two case studies and computer simulations presented in this paper indicate that cumulative subgroup analysis should be used to overcome limitations of isolated subgroup analyses in trials, and to encourage appropriate conduct, complete reporting and timely synthesis of subgroup analyses in clinical trials.

The detection of differences in effect between subgroups usually requires larger sample sizes than the evaluation of the overall treatment effect, unless the effects are in opposite directions for different subgroups. The statistical power to detect meaningful subgroup effects is unlikely to be sufficient in a single trial, and it is necessary to shift the focus from subgroup analyses in separate trials to subgroup analyses involving all related trials. Although IPD meta-analyses have been increasingly used for this purpose, cumulative subgroup analysis may detect subgroup effects much earlier than IPD meta-analyses. The early identification of important subgroup effects may help clinicians in patient care, and to inform the design and analysis of further research [48]. After the publication of IPD meta-analysis on beta blockers for heart failure in 2014 [16], there are still debates about the use of beta blockers for patients with heart failure and atrial fibrillation. For example, the results of the IPD meta-analysis was dismissed as a “retrospective subgroup analysis” in the European Society of Cardiology 2016 Guidelines for the Diagnosis and Treatment of Acute and Chronic Heart Failure [49]. Had the cumulative subgroup analysis been reported by 1999, the observed subgroup effect would have been prospectively investigated in the subsequent large scale trials, and potential mechanisms might have been better investigated.

Inflated false positive rate (type I error) is often used as a reason to restrict the conduct of multiple subgroup analyses in clinical trials. However, inflated false positive findings depend on the following two conditions: selective reporting of statistically significant results of multiple subgroup analyses, and a variety of significant subgroup effects when defined by different patient characteristics. The first problem, selective reporting, is not unique to subgroup analysis [50]. It can be addressed by complete reporting of all subgroup analyses conducted, and by clearly stating whether the subgroup analyses reported are pre-specified or post hoc. In addition, the cumulative subgroup analysis should not be stopped early when a statistically significant subgroup effect is observed, particularly at its early stage with only a few included trials. Irrespective of currently estimated subgroup effects, data from all subsequent trials conducted for various reasons should be continuously added to the cumulative subgroup analysis. The second problem may be of limited relevance in practice because it makes little sense to merge subgroup effects defined by different patient variables. For example, purely by chance, the treatment effect may be associated with age in a trial, and with different baseline variables in other trials. The results of subgroup effect by age, or by another variable, should be interpreted separately from other subgroup effects and based on pooled data from all related trials. For the same subgroup variable, the rate of false positive subgroup effect will not be inflated, and will correspond well with the statistical significance level adopted in both conventional and cumulative subgroup analyses (Tables 1 and 2). In addition, the possible harms due to false positive subgroup effects in individual trials will be minimal in practice when clinical guidelines are developed after rigorously assessing the validity of all available evidence [51].

Subgroup analyses in clinical trials may be used to test or generate hypotheses on subgroup effects [2, 52]. Given limited statistical power and lack of clear prior understanding of important subgroup variables, subgroup analyses should be generally considered as hypothesis-generating when single trials are considered in isolation. With the concept of cumulative subgroup analysis, a subgroup analysis in the first trial or a few early trials is for the purpose of hypothesis generation, but the same subgroup analysis in subsequent trials may be considered as hypothesis testing. Because it may be difficult to decide whether a subgroup analysis is hypothesis-testing or hypothesis-generating, a Bayesian approach may provide a more convenient theoretical framework for cumulative subgroup analysis [53]. Analogous to the Bayesian method of combining prior and new evidence, a cumulative subgroup analysis continuously incorporates existing information with data from a new trial.

Data on patient characteristics at baseline are routinely collected in randomised controlled trials for multiple purposes, including a description of study population, assessment of comparability of trial groups, adjusting for possible confounding factors, and conduct of subgroup analyses [54]. During the past several decades, subgroup analyses in trials have not been encouraged [10]. Consequently, data on baseline characteristics collected in trials have been under-used, or completely wasted, for the purpose of subgroup analysis. According to the recommended criteria for credible subgroup analyses [9], the number of subgroup analyses in a trial should be no more than five. Another recommended criterion for credible subgroup analyses is whether subgroup effects across related studies are consistent [9], which will be impossible to assess if the same subgroup analysis has not been conducted and reported in other related studies. The argument that only a few pre-specified subgroup analyses should be conducted in a trial conflicts with the need to compare and combine results of the same subgroup analysis from all related trials [3]. The current emphasis on avoiding false positive subgroup effects has restricted the conduct and reporting of exploratory subgroup analyses in trials, resulting in a waste of research data and missing opportunities of detecting subgroup effects that are meaningful for clinical practice or additional research [2, 48].

Inaccessibility and lack of full information are avoidable waste in biomedical research [55]. Subgroup analyses in meta-analyses using published data are often very limited or impossible due to inadequate reporting of results of subgroup analysis in trials [2, 12]. The development of IPD meta-analyses has facilitated the identification of important subgroup effects. However, trial data on subgroup effects have been wasted before the conduct of IPD meta-analyses, and continue to be wasted where IPD meta-analyses remain unavailable, although the magnitude of such waste is currently unclear. Therefore, more IPD meta-analyses of existing trials should be conducted to identify meaningful subgroup effects. In future, exploratory subgroup analyses using full data on patient characteristics at baseline should be encouraged.

Subgroup analyses in clinical trials should be conducted using appropriate statistical tests of interactions, and reporting of subgroup analyses should be complete, with sufficient information to be included in cumulative analyses. To conduct cumulative subgroup analysis, the same or similar definitions of subgroups of interest need to be adopted in related clinical trials. First, cumulative subgroup analyses should be taken into account in making decisions about data collection at the design stage. Patient subgroups could be defined according to patient baseline characteristics using data routinely collected in clinical trials. Ideally, increased sharing of trial data may enable prospective IPD meta-analysis with cumulative subgroup analyses that starts when data from the first two RCTs are available and is repeated when a new RCT is completed. Prospective IPD meta-analysis will also allow the subgroups to be defined in the same way, for example, using the same cut-points between subgroups.

The usefulness of cumulative subgroup analysis will be limited when the number of related trials is very small. Our search of PubMed (see Additional file 2 for search strategy) identified 60 IPD meta-analyses published in 2014 and only three provided sufficient data for cumulative subgroup analysis. We discussed in detail only two cases to illustrate the usefulness of cumulative subgroup analyses for clinical practice and further research. We believe that our study will inspire others to conduct more cumulative subgroup analyses using data collected in existing and future IPD meta-analyses.

## Conclusions

Without selective reporting, multiple tests of the same interaction between a treatment and a subgroup variable in related trials are not inherently associated with inflated rate of false subgroup effects. To avoid waste of research data and facilitate the early detection of important subgroup effects, subgroup analyses should be more widely conducted in clinical trials and completely reported. Subgroup analyses need to be consistently conducted across related trials using appropriate statistical methods, and their reporting should be complete, with sufficient data to be included in cumulative subgroup analysis.

## Additional files

Additional file 1: Methods of cumulative subgroup analysis. (DOCX 14 kb)
Additional file 2: Search strategy to identify cases from published individual patient data (IPD) meta-analyses. (DOCX 26 kb)
Additional file 3: Methods for computer simulations of subgroup analysis in the context of cumulative meta-analysis of randomised controlled trials. (DOCX 22 kb)

## Abbreviations

IPD: Individual patient data
OR: Odds ratio
PAH: Pulmonary arterial hypertension
RCT: Randomised controlled trial
RHR: Ratio of hazard ratios
ROR: Ratio of odds ratios
