# Supplementary material for: Cumulative subgroup analysis to reduce waste in clinical research for individualised medicine
Source: BMC Med. 2016 Dec 15;14:197. doi: 10.1186/s12916-016-0744-x (PMC5157082; doi:10.1186/s12916-016-0744-x)
Supplement: Additional file 1: — Methods of cumulative subgroup analysis. (DOCX 14 kb) [file 12916_2016_744_MOESM1_ESM.docx]

**Additional File -1**

**Methods of cumulative subgroup analysis**

For estimating overall treatment effects, cumulative meta-analysis consists of a series of repeated meta-analyses after adding data from each new trial chronologically.^1^ It can be used to reveal the contribution of individual trials to the overall estimate, and to identify the earliest time at which the pooled effect becomes statistically significant. We expanded the method of cumulative meta-analysis to investigate changes in estimates of subgroup effects over time.

Assume that patients in a trial can be categorised into two independent subgroups according to a baseline variable (e.g., female or male by sex), and the treatment effect (e.g., mean difference, or log hazard ratio) is θ_1_ for subgroup 1 and θ_2_ for subgroup 2. Then the subgroup effect (Δ) can be calculated as: Δ = θ_1_- θ_2_, and SE(Δ) =SQRT(SE(θ_1_)^2^ +SE(θ_2_)^2^), where SE(Δ), SE(θ_1_) and SE(θ_2_) are the standard error for Δ, θ_1_ and θ_2_. Statistical significance of Δ can be tested by z = Δ / SE(Δ). If the absolute value of z is greater than 1.96, the estimated Δ is considered to be statistically significant (P<0.05). Assume that *Δi* is the subgroup effect in trial *i*, and *k* trials are chronologically ordered from *i =1, 2, 3 … k*. Cumulative subgroup analysis can be performed with fixed-effect or random-effects models,^2^ in which a series of pooled subgroup effects (Δci) are calculated each time adding a new trial chronologically. The statistical significance of Δci is based on z = Δci /SE(Δci).

1. Lau J, Antman EM, Jimenez-Silva J, Kupelnick B, Mosteller F, Chalmers TC. Cumulative meta-analysis of therapeutic trials for myocardial infarction. *The New England journal of medicine* 1992; **327**(4): 248-54.

2. DerSimonian R, Laird N. Meta-analysis in clinical trials. *Controlled clinical trials* 1986; **7**(3): 177-88.
