# Supplementary material for: Cumulative subgroup analysis to reduce waste in clinical research for individualised medicine
Source: BMC Med. 2016 Dec 15;14:197. doi: 10.1186/s12916-016-0744-x (PMC5157082; doi:10.1186/s12916-016-0744-x)
Supplement: Additional file 2: — Search strategy to identify cases from published individual patient data (IPD) meta-analyses. (DOCX 26 kb) [file 12916_2016_744_MOESM2_ESM.docx]

**Additional File -2**

**Search strategy to identify cases from published individual patient data (IPD) meta-analyses**

### Inclusion criteria

- IPD meta-analyses of RCTs that evaluated effectiveness and safety of healthcare interventions
- Fully published since 2014 in English
- Provided sufficient data for cumulative subgroup analysis

### Search strategies

IPDMAs: Using the strategy below, searched PubMed on 26/09/2015:

(“meta-analysis”[TIAB] OR “systematic review”[TIAB] OR “meta-regression”[TIAB]) AND (IPD[TIAB] OR “individual patient data”[TIAB] OR “individual participant data”[TIAB] OR “patient level”[TIAB] ) AND (random*[TIAB] OR trial*[TIAB])

### PubMed Search results

- Total records: n=298
- Full text checked: n=72
- IPD meta-analyses of RCTs: n=60
- IPD meta-analyses with sufficient data for cumulative subgroup analysis n=3.[^1-3^](#_ENREF_1)
- Two case studies using data from IPD meta-analyses with sufficient data for Cumulative subgroup analysis and that included at least 10 randomised trials.[^1^](#_ENREF_1)^,^[^2^](#_ENREF_2)

### References

1. Kotecha D, Holmes J, Krum H, et al. Efficacy of beta blockers in patients with heart failure plus atrial fibrillation: an individual-patient data meta-analysis. Lancet 2014;384:2235-43.

2. Rhee RL, Gabler NB, Sangani S, Praestgaard A, Merkel PA, Kawut SM. Comparison of Treatment Response in Idiopathic and Connective Tissue Disease-associated Pulmonary Arterial Hypertension. American journal of respiratory and critical care medicine 2015;192:1111-7.

3. NSCLC. Preoperative chemotherapy for non-small-cell lung cancer: a systematic review and meta-analysis of individual participant data. Lancet 2014;383:1561-71.
