# Supplementary material for: Cumulative subgroup analysis to reduce waste in clinical research for individualised medicine
Source: BMC Med. 2016 Dec 15;14:197. doi: 10.1186/s12916-016-0744-x (PMC5157082; doi:10.1186/s12916-016-0744-x)
Supplement: Additional file 3: — Methods for computer simulations of subgroup analysis in the context of cumulative meta-analysis of randomised controlled trials. (DOCX 22 kb) [file 12916_2016_744_MOESM3_ESM.docx]

**Additional File -3:**

**Methods for computer simulations of subgroup analysis in the context of cumulative meta-analysis of randomised controlled trials**

### Computer simulation approaches

Computer simulations aims to assess true and false positive rates of conventional and cumulative subgroup analyses in trials using different input parameters in terms of the assumed subgroup effects and sample size. Simulated trials have two-arms (treatment vs control) and a binary outcome. A series of sequentially ordered RCTs were simulated. For each of the simulated trials, the computer programme randomly generated individual patients according to distributions of assumed values of input parameters, and the simulated individual patients were randomly allocated to a control and a treatment arm. Simulated patients belong to different subgroups according to four independent characteristics (X1, X2, X3 and X4) at baseline, and subgroup analyses were conducted in each simulated trial by these baseline characteristics. It is assumed that only X1 is an effect modifier, and the other three factors (X2, X3, and X4) are not truly associated with the treatment effect. The treatment effect for patients with X1 (i.e., X1=1) is greater than patients without X1 (i.e., X1=0). The simulated subgroup effect is measured by ratio of odds ratios (ROR), which is assumed to be ROR<1.0 for patients with X1, and ROR=1.0 for those without X1. For example, if ROR=0.8 for patients with X1 and OR=0.7 for patients without X1, the treatment effect for patients with X1 will be OR=0.7*0.8=0.56. We used logistic regression model with an interaction term to conduct subgroup analyses in the simulated trials.

### Input parameter values

| **Sce** | **Ntrials** | **Npts** | **OR** | **ERc** | **Hetv** | **ROR** | **pX1** | **pX2** | **pX3** | **pX4** |
| --- | --- | --- | --- | --- | --- | --- | --- | --- | --- | --- |
| 1 | 10 | 200 | 0.7 | 0.3 | 0.01 | 0.8 | 0.4 | 0.5 | 0.5 | 0.5 |
| 2 | 10 | 400 | 0.7 | 0.3 | 0.01 | 0.8 | 0.4 | 0.5 | 0.5 | 0.5 |
| 3 | 10 | 600 | 0.7 | 0.3 | 0.01 | 0.8 | 0.4 | 0.5 | 0.5 | 0.5 |
| 4 | 10 | 400 | 0.6 | 0.3 | 0.01 | 0.8 | 0.4 | 0.5 | 0.5 | 0.5 |
| 5 | 10 | 400 | 0.7 | 0.3 | 0.01 | 0.8 | 0.4 | 0.5 | 0.5 | 0.5 |
| 6 | 10 | 400 | 0.8 | 0.3 | 0.01 | 0.8 | 0.4 | 0.5 | 0.5 | 0.5 |
| 7 | 10 | 400 | 0.7 | 0.2 | 0.01 | 0.8 | 0.4 | 0.5 | 0.5 | 0.5 |
| 8 | 10 | 400 | 0.7 | 0.3 | 0.01 | 0.8 | 0.4 | 0.5 | 0.5 | 0.5 |
| 9 | 10 | 400 | 0.7 | 0.4 | 0.01 | 0.8 | 0.4 | 0.5 | 0.5 | 0.5 |
| 10 | 10 | 400 | 0.7 | 0.3 | 0 | 0.8 | 0.4 | 0.5 | 0.5 | 0.5 |
| 11 | 10 | 400 | 0.7 | 0.3 | 0.01 | 0.8 | 0.4 | 0.5 | 0.5 | 0.5 |
| 12 | 10 | 400 | 0.7 | 0.3 | 0.015 | 0.8 | 0.4 | 0.5 | 0.5 | 0.5 |
| 13 | 10 | 400 | 0.7 | 0.3 | 0.01 | 0.7 | 0.4 | 0.5 | 0.5 | 0.5 |
| 14 | 10 | 400 | 0.7 | 0.3 | 0.01 | 0.8 | 0.4 | 0.5 | 0.5 | 0.5 |
| 15 | 10 | 400 | 0.7 | 0.3 | 0.01 | 0.9 | 0.4 | 0.5 | 0.5 | 0.5 |
| 16 | 10 | 400 | 0.7 | 0.3 | 0.01 | 0.8 | 0.3 | 0.5 | 0.5 | 0.5 |
| 17 | 10 | 400 | 0.7 | 0.3 | 0.01 | 0.8 | 0.4 | 0.5 | 0.5 | 0.5 |
| 18 | 10 | 400 | 0.7 | 0.3 | 0.01 | 0.8 | 0.5 | 0.5 | 0.5 | 0.5 |

**Notes:** Sce – simulation scenarios; Ntrials – no. of simulated trials per scenario; Npts – no. of total patients in a simulated trial; OR – true odds ratio (treatment effect); ERc – event rate in the control arm; Hetv – between-study variance; ROR – ratio of odds ratios (subgroup effect); pX1 – proportion of patients with x1=1 at baseline; pX2, pX3 and pX4 – proportion of patients with X2, X3 or X4=1 at baseline.

### Simulation R –code

##===============================================

## Multiple subgroup analyses within a trial

##==============================================

SGAsim<-function() { #====Start Programme

##==============================================

## No. of simulations for a scenario

##==============================================

N.simulation <- 5000

# Output files for significant SGE for trials

Header.posit <-cbind("Sce", "TriSig1", "TriSig2", "TriSig3", "TriSig4", "TriSigR")

write.table(Header.posit, file="SigTri.out", sep="\t", quote=FALSE, append=FALSE,row.names=FALSE, col.names=FALSE)

# Output files for frequency of significant SGE for trial groups (from 0 to N.trials)

Header.freq <-cbind("Sce", "Freq", "SigX1%", "SigX2%", "SigX3%", "SigX4%")

write.table(Header.freq, file="Sigfreq.out", sep="\t", quote=FALSE, append=FALSE,row.names=FALSE, col.names=FALSE)

# Output files for cumulative analyses

Header.sigcum <-cbind("Sce", "Tri", "CumSig1", "CumSig2", "CumSig3", "CumSig4", "CumSigR")

write.table(Header.sigcum, file="SigCum.out", sep="\t", quote=FALSE, append=FALSE,row.names=FALSE, col.names=FALSE)

##================================================

## Read input data file

inPar<-read.table(file="input01.csv", header=TRUE, sep=",")

N.sce <- nrow(inPar) # No. of scenarios

##=======================================================

for(s in 1:N.sce) { # === Start for scenarios

##=======================================================

N.trials <- inPar$Ntrials[s] # No.of 2-arm trials simulated

N.pts0 <-inPar$Npts[s] # No.of pts in trt=0 arm

N.pts1 <-inPar$Npts[s] # No.of pts in trt=1 arm

N.pts2<-N.pts0+N.pts1 # Total no. of pts per trial

ER0 <- inPar$ERc[s] # Event rate in the ctr arm

OR0 <- inPar$OR[s] # Overall Odds ratio

LOR0 <- log(OR0)

het.sd <- sqrt(inPar$Hetv[s]) # Heterogeneity sd

# % patients with xi:

Px1 <- inPar$pX1[s]

Px2 <- inPar$pX2[s]

Px3 <- inPar$pX3[s]

Px4 <- inPar$pX4[s]

# Assume only X1 being effect-modifier:

ROR1 <- inPar$ROR[s]

Int1 <- vector(length=N.trials)

Int1.se <- vector(length=N.trials)

Int1.p <- vector(length=N.trials)

Int1.wt <- vector(length=N.trials)

Int2 <- vector(length=N.trials)

Int2.se <- vector(length=N.trials)

Int2.p <- vector(length=N.trials)

Int2.wt <- vector(length=N.trials)

Int3 <- vector(length=N.trials)

Int3.se <- vector(length=N.trials)

Int3.p <- vector(length=N.trials)

Int3.wt <- vector(length=N.trials)

Int4 <- vector(length=N.trials)

Int4.se <- vector(length=N.trials)

Int4.p <- vector(length=N.trials)

Int4.wt <- vector(length=N.trials)

Int1.cum <- vector(length=N.trials)

Se1.cum <- vector(length=N.trials)

Wt1.cum <- vector(length=N.trials)

CumSig1 <- vector(length=N.trials)

Int2.cum <- vector(length=N.trials)

Se2.cum <- vector(length=N.trials)

Wt2.cum <- vector(length=N.trials)

CumSig2 <- vector(length=N.trials)

Int3.cum <- vector(length=N.trials)

Se3.cum <- vector(length=N.trials)

Wt3.cum <- vector(length=N.trials)

CumSig3 <- vector(length=N.trials)

Int4.cum <- vector(length=N.trials)

Se4.cum <- vector(length=N.trials)

Wt4.cum <- vector(length=N.trials)

CumSig4 <- vector(length=N.trials)

CumSigR <- vector(length=N.trials) # For cumulative fauls SGE

Sig1.cum <- array(0,dim=c(N.simulation,N.trials))

Sig2.cum <- array(0,dim=c(N.simulation,N.trials))

Sig3.cum <- array(0,dim=c(N.simulation,N.trials))

Sig4.cum <- array(0,dim=c(N.simulation,N.trials))

SigR.cum <- array(0,dim=c(N.simulation,N.trials))

Scenar <- vector(length=N.trials)

Trials <- vector(length=N.trials)

Trial <- vector(length=N.pts2)

Ptsid <- vector(length=N.pts2)

trt <- vector(length=N.pts2)

X1 <- vector(length=N.pts2)

X2 <- vector(length=N.pts2)

X3 <- vector(length=N.pts2)

X4 <- vector(length=N.pts2)

event <- vector(length=N.pts2)

# Counting no. of trials with sig interaction

Sig1.tri <-vector(length=N.simulation)

Sig2.tri <-vector(length=N.simulation)

Sig3.tri <-vector(length=N.simulation)

Sig4.tri <-vector(length=N.simulation)

SigR.tri <-vector(length=N.simulation)

# Counting N/freq of sigSGEs for trial groups (0 to N.trials)

N.freq<-c(0:N.trials)

Sig1.fq<-vector(length=(N.trials+1))

Sig2.fq<-vector(length=(N.trials+1))

Sig3.fq<-vector(length=(N.trials+1))

Sig4.fq<-vector(length=(N.trials+1))

for(Fq in 1:(N.trials+1)) {

Sig1.fq[Fq]<-0

Sig2.fq[Fq]<-0

Sig3.fq[Fq]<-0

Sig4.fq[Fq]<-0 }

##====================================================

for(Nsim in 1:N.simulation) { #==Start==simulation

##====================================================

Sig1.tri[Nsim] <-0

Sig2.tri[Nsim] <-0

Sig3.tri[Nsim] <-0

Sig4.tri[Nsim] <-0

SigR.tri[Nsim] <-0

##================================================

## Simulate trials and patients

##------------------------------------------------

for(tri in 1:N.trials) { # Start-trials

# Add heterogeneity to logOR

# Assuming fixed effect interaction

hLOR0<- rnorm(1,LOR0,het.sd) # logOR0 with heterogeneity

hOR0 <-exp(hLOR0) # OR0 with heterogeneity

## Event rate in the trt arm

# No interaction

ER10<-(ER0*hOR0)/(1-ER0+ER0*hOR0)

# Interaction when X1=1

ER11<-(ER0*hOR0*ROR1)/(1-ER0+ER0*hOR0*ROR1)

#Start-patients in trt=0 arm:

for(j in 1:N.pts0) {

Trial[j] <- tri

Ptsid[j] <- j

trt[j] <- 0

ifelse(runif(1,0,1)<=Px1, X1[j]<-1, X1[j]<-0)

ifelse(runif(1,0,1)<=Px2, X2[j]<-1, X2[j]<-0)

ifelse(runif(1,0,1)<=Px3, X3[j]<-1, X3[j]<-0)

ifelse(runif(1,0,1)<=Px4, X4[j]<-1, X4[j]<-0)

ifelse(runif(1,0,1)<=ER0, event[j]<-1, event[j]<-0 )

} #End-patient in trt=0 arm

#Start-patients in trt=1 arm:

for(j in (1+N.pts0):N.pts2) {

Trial[j] <- tri

Ptsid[j] <- j

trt[j] <- 1

ifelse(runif(1,0,1)<=Px1, X1[j]<-1, X1[j]<-0)

ifelse(runif(1,0,1)<=Px2, X2[j]<-1, X2[j]<-0)

ifelse(runif(1,0,1)<=Px3, X3[j]<-1, X3[j]<-0)

ifelse(runif(1,0,1)<=Px4, X4[j]<-1, X4[j]<-0)

ifelse(runif(1,0,1)<=ER10,event[j]<-1,event[j]<-0) # No interaction

if(X1[j]==1) {

ifelse(runif(1,0,1)<=ER11,event[j]<-1,event[j]<-0) }

} #End-patient in trt=1 arm

#========================================================

# Subgroup analysis using logistic regression analysis

#-------------------------------------------------------

# For subgroup by X1:

#-----------------------------

sga.1 <- glm(event ~ trt +X1 +trt:X1, family="binomial")

# Extract interaction coefficients

Int1[tri] <- summary(sga.1)$coefficients[4,1]

Int1.se[tri] <- summary(sga.1)$coefficients[4,2]

Int1.p[tri] <- summary(sga.1)$coefficients[4,4]

Int1.wt[tri] <- 1/(Int1.se[tri]^2)

ifelse((Int1.p[tri]<=0.0500), sig1<-1, sig1<-0)

Sig1.tri[Nsim] <- Sig1.tri[Nsim]+sig1

#-------------------------------------------------------

# For subgroup by X2:

#-----------------------------

sga.2 <- glm(event ~ trt +X2 +trt:X2, family="binomial")

# Extract interaction coefficients

Int2[tri] <- summary(sga.2)$coefficients[4,1]

Int2.se[tri] <- summary(sga.2)$coefficients[4,2]

Int2.p[tri] <- summary(sga.2)$coefficients[4,4]

Int2.wt[tri] <- 1/(Int2.se[tri]^2)

ifelse((Int2.p[tri]<=0.0500), sig2<-1, sig2<-0)

Sig2.tri[Nsim] <- Sig2.tri[Nsim]+sig2

#-------------------------------------------------------

# For subgroup by X3:

#-----------------------------

sga.3 <- glm(event ~ trt +X3 +trt:X3, family="binomial")

# Extract interaction coefficients

Int3[tri] <- summary(sga.3)$coefficients[4,1]

Int3.se[tri] <- summary(sga.3)$coefficients[4,2]

Int3.p[tri] <- summary(sga.3)$coefficients[4,4]

Int3.wt[tri] <- 1/(Int3.se[tri]^2)

ifelse((Int3.p[tri]<=0.0500), sig3<-1, sig3<-0)

Sig3.tri[Nsim] <- Sig3.tri[Nsim]+sig3

#-------------------------------------------------------

# For subgroup by X4:

#-----------------------------

sga.4 <- glm(event ~ trt +X4 +trt:X4, family="binomial")

# Extract interaction coefficients

Int4[tri] <- summary(sga.4)$coefficients[4,1]

Int4.se[tri] <- summary(sga.4)$coefficients[4,2]

Int4.p[tri] <- summary(sga.4)$coefficients[4,4]

Int4.wt[tri] <- 1/(Int4.se[tri]^2)

ifelse((Int4.p[tri]<=0.0500), sig4<-1, sig4<-0)

Sig4.tri[Nsim] <- Sig4.tri[Nsim]+sig4

#-----------------------------------------------

# Any sig SGE by X2, X3 or X4

#-----------------------------------------------

SigR <- sig2+sig3+sig4

if(SigR>=1) SigR.tri[Nsim]<-SigR.tri[Nsim]+1

##=====================================================

# Cumulative MA for Interaction X1

#----------------------------------

if(tri==1) {

Int1.cum[tri] <- Int1[tri]

Se1.cum[tri] <- Int1.se[tri] }

if(tri>1) {

Int1.cum[tri]<-(Int1.cum[tri-1]*Wt1.cum[tri-1]+Int1[tri]*Int1.wt[tri])/

(Wt1.cum[tri-1]+Int1.wt[tri])

Se1.cum[tri] <- sqrt(1/(Wt1.cum[tri-1]+Int1.wt[tri])) }

Wt1.cum[tri] <- 1/(Se1.cum[tri]^2)

Z1.cum <- abs(Int1.cum[tri]/Se1.cum[tri])

P1.cum <- 2*(1-pnorm(Z1.cum))

ifelse((P1.cum<=0.0500), cumsig<-1, cumsig<-0)

Sig1.cum[Nsim,tri] <-cumsig

#----------------------------------

# Cumulative MA for Interaction X2

#----------------------------------

if(tri==1) {

Int2.cum[tri] <- Int2[tri]

Se2.cum[tri] <- Int2.se[tri] }

if(tri>1) {

Int2.cum[tri]<-(Int2.cum[tri-1]*Wt2.cum[tri-1]+Int2[tri]*Int2.wt[tri])/

(Wt2.cum[tri-1]+Int2.wt[tri])

Se2.cum[tri] <- sqrt(1/(Wt2.cum[tri-1]+Int2.wt[tri])) }

Wt2.cum[tri] <- 1/(Se2.cum[tri]^2)

Z2.cum <- abs(Int2.cum[tri]/Se2.cum[tri])

P2.cum <- 2*(1-pnorm(Z2.cum))

ifelse((P2.cum<=0.0500), cumsig<-1, cumsig<-0)

Sig2.cum[Nsim,tri] <-cumsig

#----------------------------------

# Cumulative MA for Interaction X3

#----------------------------------

if(tri==1) {

Int3.cum[tri] <- Int3[tri]

Se3.cum[tri] <- Int3.se[tri] }

if(tri>1) {

Int3.cum[tri]<-(Int3.cum[tri-1]*Wt3.cum[tri-1]+Int3[tri]*Int3.wt[tri])/

(Wt3.cum[tri-1]+Int3.wt[tri])

Se3.cum[tri] <- sqrt(1/(Wt3.cum[tri-1]+Int3.wt[tri])) }

Wt3.cum[tri] <- 1/(Se3.cum[tri]^2)

Z3.cum <- abs(Int3.cum[tri]/Se3.cum[tri])

P3.cum <- 2*(1-pnorm(Z3.cum))

ifelse((P3.cum<=0.0500), cumsig<-1, cumsig<-0)

Sig3.cum[Nsim,tri] <-cumsig

#----------------------------------

# Cumulative MA for Interaction X4

#----------------------------------

if(tri==1) {

Int4.cum[tri] <- Int4[tri]

Se4.cum[tri] <- Int4.se[tri] }

if(tri>1) {

Int4.cum[tri]<-(Int4.cum[tri-1]*Wt4.cum[tri-1]+Int4[tri]*Int4.wt[tri])/

(Wt4.cum[tri-1]+Int4.wt[tri])

Se4.cum[tri] <- sqrt(1/(Wt4.cum[tri-1]+Int4.wt[tri])) }

Wt4.cum[tri] <- 1/(Se4.cum[tri]^2)

Z4.cum <- abs(Int4.cum[tri]/Se4.cum[tri])

P4.cum <- 2*(1-pnorm(Z4.cum))

ifelse((P4.cum<=0.0500), cumsig<-1, cumsig<-0)

Sig4.cum[Nsim,tri] <-cumsig

#-------------------------------------------------------------------------

# Any false cumulative sig SGE for X2, X3 and X4

#-------------------------------------------------------------------------

SigRcum<-Sig2.cum[Nsim,tri]+Sig3.cum[Nsim,tri]+Sig4.cum[Nsim,tri]

ifelse(SigRcum>=1, SigR.cum[Nsim,tri]<-1, SigR.cum[Nsim,tri]<-0)

#-----------------------------------------------------------------------

} # =====End trials 'tri' #

#-----------------------------------------------------------------------

# Count no. of sigSGEs for X1-X4 (from 0 to N.trials)

for(Fq in 1:(N.trials+1)) {

nFq <-(Fq-1)

if(Sig1.tri[Nsim]==nFq) Sig1.fq[Fq]<-Sig1.fq[Fq]+1

if(Sig2.tri[Nsim]==nFq) Sig2.fq[Fq]<-Sig2.fq[Fq]+1

if(Sig3.tri[Nsim]==nFq) Sig3.fq[Fq]<-Sig3.fq[Fq]+1

if(Sig4.tri[Nsim]==nFq) Sig4.fq[Fq]<-Sig4.fq[Fq]+1 }

#-----------------------------------------------------------------------

} #====End simulations 'Nsim' #

#-----------------------------------------------------------------------

# Rate of positive SGE per trial (no. of trials with sig-SGE/all simulated trials)

# Sig1.pos - true positive (power); SigR.pos - false positive rate

Sig1.pos <- round(mean(Sig1.tri)/N.trials, digits=4)

Sig2.pos <- round(mean(Sig2.tri)/N.trials, digits=4)

Sig3.pos <- round(mean(Sig3.tri)/N.trials, digits=4)

Sig4.pos <- round(mean(Sig4.tri)/N.trials, digits=4)

SigR.pos <- round(mean(SigR.tri)/N.trials, digits=4)

out.sigtri <- cbind(s, Sig1.pos, Sig2.pos, Sig3.pos, Sig4.pos, SigR.pos)

write.table(out.sigtri, file="SigTri.out", sep="\t", quote=FALSE, append=TRUE,col.names=FALSE, row.names=FALSE)

# Frequency of sigSGEs for each group of trials (from 0 to N.trials)

for(Fq in 1:(N.trials+1)) {

Sig1.fq[Fq]<-round(100*Sig1.fq[Fq]/N.simulation, digits=4)

Sig2.fq[Fq]<-round(100*Sig2.fq[Fq]/N.simulation, digits=4)

Sig3.fq[Fq]<-round(100*Sig3.fq[Fq]/N.simulation, digits=4)

Sig4.fq[Fq]<-round(100*Sig4.fq[Fq]/N.simulation, digits=4) }

out.Sigfreq <- cbind(s, N.freq, Sig1.fq, Sig2.fq, Sig3.fq, Sig4.fq)

write.table(out.Sigfreq, file="Sigfreq.out", sep="\t", quote=FALSE, append=TRUE,col.names=FALSE, row.names=FALSE)

# Cumulative rate of positive SGE by "year":

for(tri in 1:N.trials) {

CumSig1[tri]<-round(mean(Sig1.cum[,tri]), digits=4)

CumSig2[tri]<-round(mean(Sig2.cum[,tri]), digits=4)

CumSig3[tri]<-round(mean(Sig3.cum[,tri]), digits=4)

CumSig4[tri]<-round(mean(Sig4.cum[,tri]), digits=4)

CumSigR[tri]<-round(mean(SigR.cum[,tri]), digits=4)

Scenar[tri] <- s

Trials[tri] <-tri }

out.sigcum <- cbind(Scenar, Trials, CumSig1, CumSig2, CumSig3, CumSig4, CumSigR)

write.table(out.sigcum, file="SigCum.out", sep="\t", quote=FALSE, append=TRUE,col.names=FALSE, row.names=FALSE)

#------------------------------------------------------

} #===End scenario 's' #

#------------------------------------------------------

} #====End programme #

#------------------------------------------------------
